# Soluble Epoxide Hydrolase Blockade after Stroke Onset Protects Normal but Not Diabetic Mice

**DOI:** 10.3390/ijms22115419

**Published:** 2021-05-21

**Authors:** Catherine M. Davis, Wenri H. Zhang, Elyse M. Allen, Thierno M. Bah, Robert E. Shangraw, Nabil J. Alkayed

**Affiliations:** 1 Department of Anesthesiology & Perioperative Medicine, Oregon Health & Science University, 3181 S.W. Sam Jackson Pk. Rd., UHN-2, Portland, OR 97239-3098, USA; davis@ohsu.edu (C.M.D.); zhangw@ohsu.edu (W.H.Z.); alleel@ohsu.edu (E.M.A.); baht@ohsu.edu (T.M.B.); shangraw@ohsu.edu (R.E.S.); 2 The Knight Cardiovascular Institute, Oregon Health & Science University, 3181 S.W. Sam Jackson Pk. Rd., UHN-2, Portland, OR 97239-3098, USA

**Keywords:** type 2 diabetes mellitus, sEH, stroke

## Abstract

Soluble epoxide hydrolase (sEH) is abundant in the brain, is upregulated in type 2 diabetes mellitus (DM2), and is possible mediator of ischemic injury via the breakdown of neuroprotective epoxyeicosatrienoic acids (EETs). Prophylactic, pre-ischemic sEH blockade with 4-[[*trans*-4-[[(tricyclo[3.3.1.13,7]dec-1-ylamino)carbonyl]amino]cyclohexyl]oxy]-benzoic acid (tAUCB) reduces stroke-induced infarct in normal and diabetic mice, with larger neuroprotection in DM2. The present study tested whether benefit occurs in normal and DM2 mice if tAUCB is administered after stroke onset. We performed 60 min middle cerebral artery occlusion in young adult male C57BL mice divided into four groups: normal or DM2, with t-AUCB 2 mg/kg or vehicle 30 min before reperfusion. Endpoints were (1) cerebral blood flow (CBF) by laser Doppler, and (2) brain infarct at 24 h. In nondiabetic mice, t-AUCB reduced infarct size by 30% compared to vehicle-treated mice in the cortex (31.4 ± 4 vs. 43.8 ± 3 (SEM)%, respectively) and 26% in the whole hemisphere (26.3 ± 3 vs. 35.2 ± 2%, both *p* < 0.05). In contrast, in DM2 mice, tAUCB failed to ameliorate either cortical or hemispheric injury. No differences were seen in CBF. We conclude that tAUCB administered after ischemic stroke onset exerts brain protection in nondiabetic but not DM2 mice, that the neuroprotection appears independent of changes in gross CBF, and that DM2-induced hyperglycemia abolishes t-AUCB-mediated neuroprotection after stroke onset.

## 1. Introduction

Rapid brain reperfusion, either by pharmacologic thrombolysis or mechanical thrombectomy, is the definitive treatment for acute ischemic stroke if not contraindicated. Outcomes from aggressive reperfusion have generally been good, but adjunct intravascular therapeutic options to limit ischemia-reperfusion injury are limited [1,2].

Patients with insulin-resistant diabetes mellitus type 2 (DM2), are at 2-fold increased risk for suffering acute ischemic stroke, particularly of large vessel etiology [3,4,5]. DM2 patients are consequently over-represented in the acute ischemic stroke population, such that half of patients present with hyperglycemia, most of which is secondary to comorbid DM2 [6,7,8,9,10]. Furthermore, once ischemic stroke has occurred, a hyperglycemic patient is at increased risk of a poor neurological recovery, whether the immediate treatment for stroke is pharmacological thrombolysis [11,12], mechanical thrombectomy [13,14,15] or conservative therapy [10,16,17].

Soluble epoxide hydrolase (sEH), a product of the *EPHX2* gene, is abundant in the brain and is a possible mediator for ischemia-reperfusion injury via its breakdown of neuroprotective epoxyeicosatrienoates (EETs) [18,19,20,21,22]. We have previously shown that both insulin-resistant prediabetes, which leads to DM2, or the much less common insulinopenic diabetes mellitus type 1 (DM1) both upregulate *EPHX2* and increase sEH protein expression [23,24]. Prophylactic subchronic blockade of sEH with tAUCB, which acts to restore brain EET concentration and reduces brain infarct size after transient middle cerebral artery occlusion (MCAO) in mice, an effect elicited in normal mice but even more robust in the setting of DM2 or DM1, leading to the possibility that the “penalty of diabetes” in stroke could be ameliorated by effective sEH blockade [23,24].

The present study was designed to test whether administration of sEH blockade after that onset of ischemia, i.e., once stroke had initiated, can protect the brain from ischemia-reperfusion in both non-diabetic and DM2 mice.

## 2. Results

### 2.1. tAUCB Reduces Infarct Size in Non-Diabetic Mice

**Figure 1:**
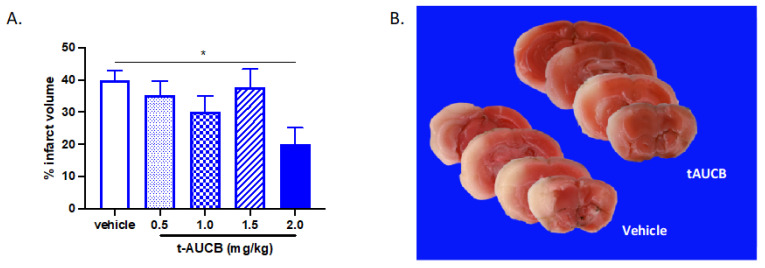
tAUCB administration at reperfusion reduces infarct volume. Mice were given 0.5, 1.0, 1.5 or 2.0 mg/kg tAUCB i.p. at the time of vessel reperfusion, hemispheric infarct was assessed 24 h later by TTC. (**A**) The highest dose tested, 2.0 mg/kg, reduced infarct (* *p* < 0.05, 2.0 mg/kg vs. vehicle); lower doses of tAUCB did not significantly affect infarct volume. *n* = 4–7/group. (**B**) Representative TTC images coronal brain sections from mice treated with 2.0 mg/kg tAUCB dose.

**Figure 2:**
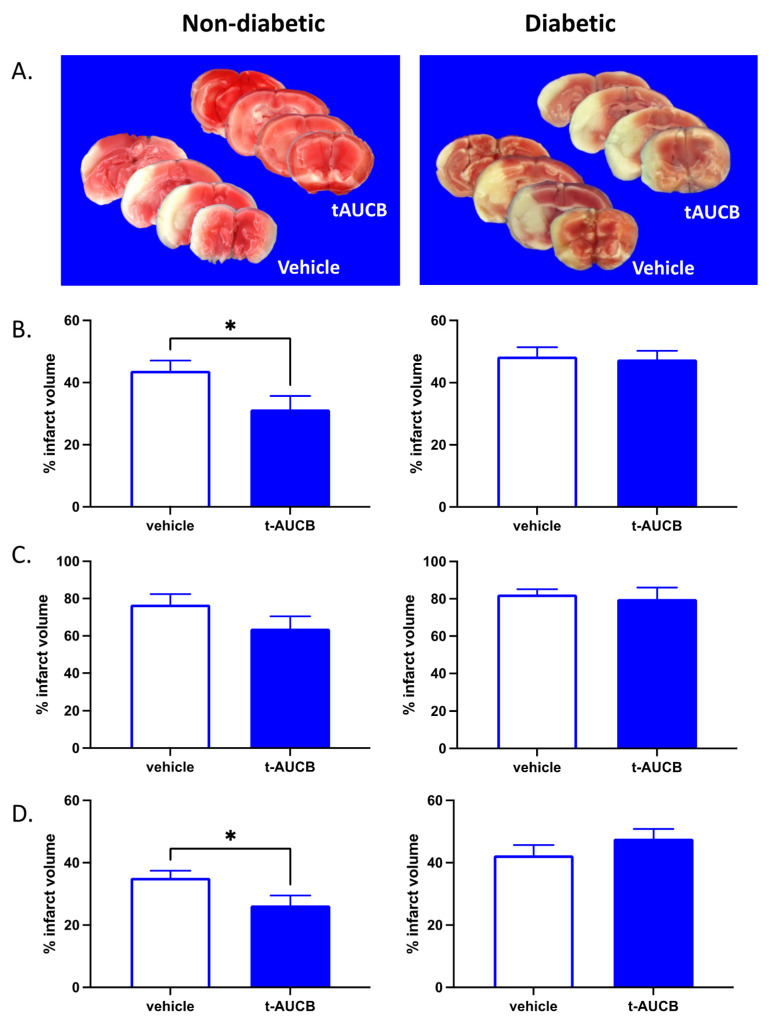
tAUCB administered during vessel occlusion reduces infarct volume in non-diabetic, but not in diabetic mice. tAUCB, 2 mg/kg, was administered i.p. 30 min into 1 h MCA occlusion, infarct was assessed by TTC 24 h later. (**A**) Representative TTC images of coronal sections from non-diabetic (**left**) and diabetic (**right**) mouse brains. Infarct volume in (**B**) cortex, (**C**) caudate putamen, and (**D**) hemisphere of non-diabetic (**left**) and diabetic (**right**) mice, *n* = 7–8, * *p* < 0.05.

We first determined whether tAUCB administration at the time of vessel reperfusion is protective following MCAO. Four doses of tAUCB were tested: 0.5, 1.0, 1.5 and 2.0 mg/kg, administered i.p. at the same time as filament removal from MCA (Figure 1). We found that 2.0 mg/kg reduces hemispheric infarct from 40.03 ± 2.96% in vehicle-treated mice to 20.16 ± 5% in tAUCB-treated mice (*p* < 0.05); lower doses did not significantly alter infarct size (ordinary one-way ANOVA with multiple comparisons tests, *n* = 4–7 per dose). We subsequently used this optimal dose of 2 mg/kg tAUCB to determine whether sEH blockade during vessel occlusion would be protective for non-diabetic and diabetic mice. Type 2 diabetes (DM2) was induced by a combination of a high-fat diet with nicotinamide (NA) and streptozotocin (STZ) treatment, as described in methods. When administered i.p. mid-way through 60 min MCAO, tAUCB was protective for non-diabetic mice, reducing hemispheric infarct from 35.19 ± 2.26% in vehicle- to 26.34 ± 3.16% in tAUCB-treated mice (*p* < 0.05, unpaired *t*-test); however, no protection was evident in DM2 mice (Figure 2; *n* = 7–8/group).

### 2.2. tAUCB Does Not Alter Blood Glucose Concentration in Diabetic Mice

**Figure 3:**
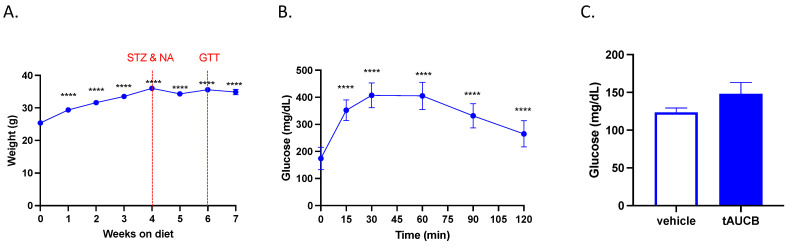
tAUCB does not alter blood glucose concentration in diabetic mice. Mice on high-fat diet injected with NA and STZ develop a diabetic phenotype: (**A**) Mice were placed on a high-fat diet for 7 weeks, and injected with NA and STZ (indicated by first vertical red line) to induce type 2 diabetes, body weight increased following 1 week on high-fat diet, and persisted throughout (*p* < 0.0001 vs. week 0, *n* = 16). (**B**) After 6 weeks on high-fat diet (indicated by second vertical red line), and 2 weeks following NA and STZ injections, glucose tolerance testing revealed hyperglycemia at baseline (time 0), and impaired glucose clearance with blood glucose concentration remaining elevated 2 h after ingestion of 2 g/kg glucose (**** *p* < 0.0001 vs. time 0, *n* = 16). (**C**) Blood glucose concentration measured 24 h following tAUCB administration and MCAO show no difference between tAUCB and vehicle groups in DM2 mice (*n*/*s*, *n* = 8/group).

We previously demonstrated that subchronic (7 day) tAUCB treatment reduces blood glucose concentration after stroke [24]. We therefore investigated whether the single injection of tAUCB during vessel occlusion used in this study also affected blood glucose levels. First, we confirmed the presence of DM2 in mice one week prior to MCAO, as indexed by fasting hyperglycemia in the DM2 cohort. Blood glucose concentration was 174.06 ± 10.34 mg/dL, elevated above established levels of approximately 100 mg/dL in non-diabetic mice, as previously published [24]. When subjected to a glucose tolerance test (GTT), blood glucose concentration was persistently increased above the baseline even at 2 h post-glucose ingestion, indicating impaired glucose clearance (Figure 3B; *p* < 0.0001, one-way ANOVA with Dunnett’s multiple comparisons test, *n* = 16). Subsequently, blood glucose concentration at 24 h post-MCAO, immediately before sacrifice, was not different between vehicle and tAUCB groups (Figure 3C; *t*-test, *n* = 8/group).

### 2.3. tAUCB Selectively Increases Plasma 14,15-EET Concentration

**Figure 4:**
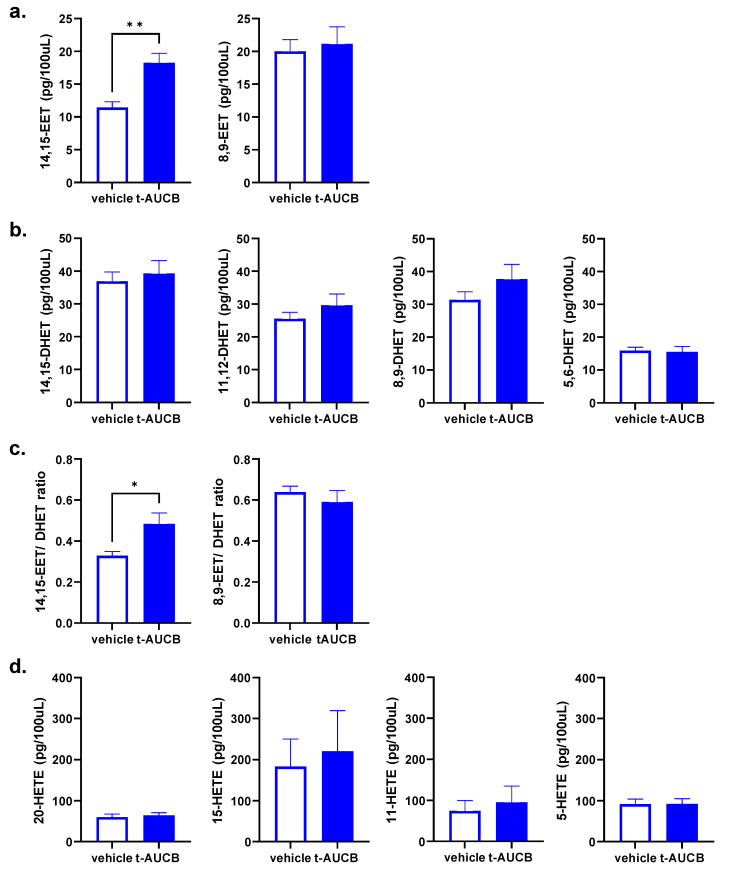
tAUCB selectively increases plasma 14,15-EET and 14,15-EET:DHET ratio in diabetic mice. Plasma concentrations of (**a**) EET, (**b**) DHET, (**c**) EET:DHET ratio and (**d**) HETE regioisomers assessed by LC-MS/MS 24 h following tAUCB administration and MCAO. Plasma 14,15-EET concentration and 14,15-EET:DHET ratio, are elevated in the tAUCB group compared to vehicle (** *p* < 0.01, * *p* < 0.05, *n* = 7–8/group).

The preferred EETs regioisomer as a substrate for sEH is 14,15-EET. Blockade of sEH by tAUCB inhibits the degradation of 14,15-EET to its metabolic product 14,15-dihydroxyeicosatrienoate (14,15-DHET). We therefore investigated the effect of sEH blockade on circulating 14,15-EET levels, as well as other regioisomers, in diabetic mice by liquid chromatography-tandem mass spectrometry (LC-MS/MS) (Figure 4). Plasma collected at the time of sacrifice, 24 h post-MCAO, demonstrates that tAUCB increases circulating 14,15-EET, as well as the 14,15-EET/DHET ratio specifically, without altering other EETs, HETEs and DHETs. The plasma concentration of 14,15-EET was increased from 11.48 ± 0.84 in vehicle-treated to 18.29 ± 1.42 pg/100 uL in tAUCB-treated mice (*p* < 0.01, unpaired *t*-test), while not affecting 8,9-EET concentration; 11,12-EET, while present, was below levels of detection in both vehicle and tAUCB groups (Figure 4a; *n* = 7–8/group). DHET and HETE concentrations were unaltered by tAUCB treatment (Figure 4b,d).

### 2.4. CBF Is Unaltered by tAUCB in Immediate Peri-Ischemia Period

**Figure 5:**
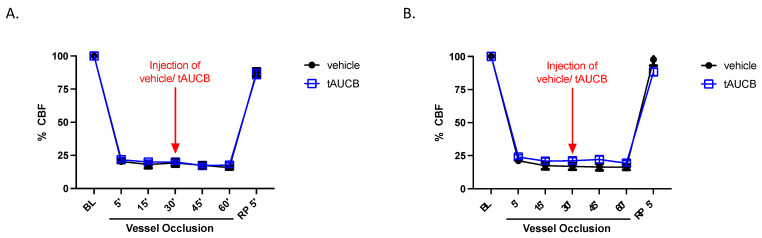
CBF is unaltered by tAUCB. CBF, assessed by laser Doppler probe, during and immediately after MCA occlusion does not differ between vehicle or tAUCB treated mice in either (**A**) non-diabetic, or (**B**) diabetic mice (BL, baseline; RP5’, 5 min reperfusion; *n*= 7–8/group).

We have previously shown that subchronic (7 day) tAUCB treatment improves reperfusion immediately following MCAO in DM2 mice, compared to vehicle [24]. Therefore, CBF was also assessed in this current paradigm of single injection during and immediately after vessel occlusion. Figure 5 shows that relative cerebrocortical perfusion in the MCA territory, measured by laser Doppler, is not different between vehicle and tAUCB groups in either non-diabetic or diabetic mice, 5 min post-reperfusion (2-way ANOVA, *n* = 7–8/group), suggesting that t-AUCB does not alter ischemic severity or the degree of reperfusion after filament withdrawal.

## 3. Discussion

The present study demonstrates that post-ischemic administration of tAUCB to block sEH exerts brain protection in non-diabetic mice, but fails to offer any neuroprotection in mice with type 2 diabetes (DM2), in a transient focal cerebral ischemia model. The timing of tAUCB administration in our study mimics a real-life clinical application of a stroke therapeutic, where it is administered to patients while the obstructing clot is still in place. The standard of care for stroke is clot lysis using tissue plasminogen activator (tPA) or clot removal using endovascular thrombectomy. Neuroprotectants such as sEH inhibitors can be given either before, in conjunction with, or directly after clot retrieval or lysis to protect neural tissue, suppress inflammation and improve microvascular collateral blood flow. By protecting tissue until clot is removed or lysed, this so-called “bridging therapy” extends the time window for when standard of care can be administered [25].

In our non-diabetic mice, the tAUCB-mediated 30% reduction in cortical infarct size comports with the preservation of EET-mediated neuroprotection against ischemia-reperfusion injury, which is new because tAUCB was not administered until after onset of focal ischemia. This observation fits the concept that sEH blockade in the setting of acute ischemic stroke could potentially serve as an adjunct therapy at reperfusion to improve outcome from stroke. It is noteworthy that immediate gross macrovascular cerebral blood flow (as measured by laser-Doppler technique, Figure 5) was not different between the tAUCB and control groups, at least within the early peri-ischemic time frame. This indicates that the beneficial effect of tAUCB does not seem to be mediated by changes in immediate post-reperfusion regional blood flow.

In our DM2 mice, the trend towards larger MCAO infarct size compared to non-diabetic counterparts (48 vs. 44% of cortex, and 42 vs. 35% of hemisphere affected, resp. at 24 h postischemia), although failing to achieve statistical significance due to small sample size, is consistent with the finding of DM2-induced larger infarct size in previous observations in our laboratory [24] and with clinical MRI imaging [26]. The failure of tAUCB to reduce brain injury in DM2 mice when administered post-ischemia contrasts with our findings in non-diabetic mice, and also with the observed ability of tAUCB to protect the brain in DM2 mice at least as effectively as that in non-diabetic mice when administered pre-emptively [24]. Our current finding that tAUCB administered after onset of ischemia failed to elicit neuroprotection in DM2 mice was unexpected but not unprecedented, as hyperglycemia also eliminates ischemic preconditioning protection against ischemia-reperfusion in myocardium [27,28], and also in incubated kidney glomerular endothelial cells [29]. The mechanism for this action requires further evaluation and could involve a common pathway. There are several possible explanations. It is possible that tAUCB failed to inhibit sEH activity when administered post-ischemia. However, this is not supported by our observation that plasma EET concentration in DM2 mice treated with tAUCB was doubled at 24 h post stroke. Additionally, the observed brain protection in non-diabetic mice indicates that the time course of tAUCB on sEH activity should have had some effect on brain injury in DM2 mice. We have previously demonstrated that pre-diabetes insulin resistance leading to DM2 upregulates the *EPHX2* gene which codes for sEH and consequently increases sEH content in mouse brain [24]. It is well established that sEH activity inactivates neuroprotective EETs [18]. However, the timeline for dynamics regulating EETs’ activity specifically in the brain are unknown. It is possible that that chronic DM2-induced hyperglycemia and upregulation of sEH before the onset of ischemia markedly decreased EET concentrations in the brain which could not rapidly be restored by a single dose of tAUCB present in the circulation at the time of reperfusion. The speed of onset of sEH inhibition may not have been fast enough to restore EET concentration in the vulnerable brain watershed at reperfusion. It is also possible that a downstream defect interferes with the ability of EETs to protect the brain in a hyperglycemic environment, although this is not supported by the ability of pre-emptive tAUCB (and EETs) to protect brain when administered pre-emptively even with a very modest effect on prevailing hyperglycemia in DM2 mice [24]. This hypothesis could be tested by direct administration of EETs or a more stable EETs analog at the time of reperfusion.

We have previously shown that tAUCB, when administered subchronically before MCAO ischemia to inhibit sEH as a preventative agent, provides protection against focal transient ischemic brain injury in mice with type 2 or much less common type 1 DM, an effect even larger than observed in normoglycemic mice [23,24]. Some of the protection observed in type 2 (but not type 1) DM mice pre-emptively treated with tAUCB before ischemia could have been that tAUCB improved, to modest extent, glycemic status before and at the time of ischemia [24], an effect precluded when tAUCB treatment was delayed until after ischemia onset, as in the present study.

Half of patients (40–57%) presenting with new onset ischemic stroke are hyperglycemic at time of presentation [6,7,8,10], with type 2 diabetes the single largest contributor to the hyperglycemia. In fact, a recent meta-analysis found that the prevalence of DM2 in the ischemic stroke population was 33% [9]. Hyperglycemic stroke patients can be stratified into groups of known DM2 diagnosis, previously unknown new DM2 diagnosis, and nondiabetic patients exhibiting “stress hyperglycemia” uniquely related to the ischemic event itself [10,16]. Hyperglycemia and poor glucose tolerance are associated with poor clinical outcome from stroke [10,30]. The largest detrimental effect of hyperglycemia seems to occur in patients without a history of (or treatment of) DM2 [30], followed by those in who the diagnosis of DM2 is made during the admission for stroke [10,30]. Among hyperglycemic patients, the “best” outcome is seen in patients in whom the diagnosis of DM2 was made and treatment was initiated before onset of the stroke. The worse outcome from stroke in a hyperglycemic setting occurred when the reperfusion treatment for stroke was pharmacologic thrombolysis [11,12], mechanical thrombectomy [13,14,15], or expectant therapy [10,16,17]. Our MCAO model most closely resembles the technique of mechanical thrombectomy, which in clinical practice is the most recent addition to the therapeutic options for stroke. The molecular mechanism(s) underlying increased vulnerability to transient focal ischemia during evolution of DM2 are poorly understood but appear to include increased vascular inflammation, tissue lactic acidosis, pathologic platelet aggregation, cellular oxidative stress and impaired mitochondrial function [26,31,32,33,34,35]. At the time of reperfusion, limited therapeutic options are currently available, other than reasonable glycemic standard-of-care control with insulin. It is worth noting that increased risk of stroke in patients with DM2 is substantially eliminated when, among other controllable variables, good glycemic control leading to a glycated hemoglobin (Hb1AC) of <7.0% is maintained, in a study of 1.5 million patients [5]. Rawshani et al. also showed that poor glycemic control, as indexed by Hb1AC > 7% concentration, was the strongest predictor of stroke among DM2 patients [5]. However, the Rawshani et al. study was not aimed at determining outcome from stroke. Tight insulin-mediated glycemic control post-stroke in hyperglycemic patients failed to improve clinical outcomes from acute ischemic stroke in two large clinical trials, GIST-UK and SHINE [36,37]. The use of sulfonylurea hypoglycemic agents, while widely prescribed in DM2 patients for baseline glycemic control, may be contraindicated in clinical setting post cerebral ischemia because their mechanism of action, the inhibition of pancreatic beta-cell KATP channels to augment insulin secretion, may concurrently interfere with cerebral ischemic protection by a mechanism that requires neuronal KATP channel activation [38]. There is limited experience with newer hypoglycemic agents such as sodium-glucose co-transporter inhibitor (SGLT2i, e.g., empagliflozin [39]), glucagon-like peptide-1 receptor agonist (GLP-1RA, e.g., liraglutide, dulaglutide [40,41] or peroxisome proliferator-activated receptor agonists (PPAR-A) which reduce systemic insulin resistance (e.g., pioglitazone [42]) in terms of neuroprotection post-stroke, as their investigations have focused on demonstrating the prevention of stroke or other long-term sequalae rather than improving the outcome from it [39,40,41,42]. In the one study which examined the effect of GLP-1A stimulation post-ischemia, treatment with linagliptin was only initiated at 3 days after transient MCAO stroke and showed some positive effects on chronic neurological outcome, while sulfonylurea treatment offered no improvement vs. control [43].

Protection of the brain using tAUCB to inhibit sEH occurs via permissive accumulation of neuroprotective EETs, which are otherwise broken down and inactivated by sEH [44]. EETs directly protect by means of several potentially complementary mechanisms: they enhance vasodilation and cerebral blood flow [45], facilitate cerebrovascular integrity and microvascular density [46], inhibit platelet adhesion [47], reduce inflammation [48], reduce oxygen free radicals [49], slow apoptosis, and activate protective signal transduction [45,50].

A limitation of the present study is that we did not measure microcirculation (as opposed to gross carotid arterial inflow) or an extended timeline for carotid flow post reperfusion in the 24 h before sacrifice. Another limitation is that we did not assess functional neurologic outcomes of sEH inhibition following stroke. The reasons for this are two-fold; firstly, tAUCB did not alter infarct size at 24 h after diabetic stroke, making it unlikely that it would have a positive impact on long-term outcome, the most important determinant of which is the size and severity of the initial injury. Secondly, while sEH inhibition may still work as a therapy to promote long-term recovery (as opposed to acute neuroprotection) after diabetic stroke, our study was not designed to test that hypothesis. To determine if tAUCB promotes long-term recovery, it would need to be administered at a delayed time point, such as 3 or 7 days post-MCAO, when the initial injury has subsided and infarct size has been established. A third limitation is that we did not establish an exact time course for EETs or sEH protein concentrations in the 24 h interval between the onset of reperfusion and the assessment of infarct size, and details of that 24 h window could be important. A fourth limitation is that we did not measure EET concentrations in the brain itself at any time. Finally, we have not established a dose–response curve for tAUCB on either sEH activity or EET concentrations, and administered tAUCB based on body weight in all mice, such that DM2 mice received a larger absolute tAUCB dose, but it is not clear whether a larger or smaller dose than utilized could have changed the outcome in the setting of DM2. In fact, the 2.0 mg/kg dose used in the current study is double that used in our previous study assessing subchronic tAUCB treatment prior to stroke (1.0 mg/kg), which was effective in reducing infarct in diabetic mice [24].

To summarize, we found that sEH blockade with tAUCB functionally administered at carotid reperfusion limits brain infarct size after MCAO in normal mice, but this post-ischemic neuroprotection is abolished by uncontrolled DM2. This appears to limit the potential utility of tAUCB in the treatment of acute ischemic stroke in the setting of DM2, although it leaves unanswered the possible consequence of administering a higher dose of tAUCB or of directly administering EETs or an EETs analog at reperfusion. It also remains to be tested whether tAUCB exerts neuroprotection in nondiabetic mice which exhibit “stress hyperglycemia” at the time of stroke onset or in setting of DM2 with better regulated glycemia.

## 4. Materials and Methods

### 4.1. Animals

This study was conducted in accordance with the National Institute of Health’s guidelines for the care and use of animals in research, and protocols were approved by the Institutional Animal Care and Use Committee at Oregon Health and Science University, Portland, OR, USA (protocol number TR01_IP00000147, approved 21/09/2018). Reporting of results conforms to ARRIVE 2.0 (Animal Research: Reporting on In Vivo Experiments) guidelines [51]. Adult male wild-type C57BL/6J (The Jackson Laboratory, Bar Harbor, ME, USA) mice were used, arriving at 9 weeks of age and subjected to MCAO at 16–17 weeks of age.

### 4.2. Induction of Type 2 Diabetes

Upon arrival, 9-week-old male C57BL/6J mice were acclimatized to the animal facility for one week and subsequently placed on a high-fat (60% fat) diet (D12492i, Research Diets, Inc., New Brunswick, NJ, USA). Weight was tracked weekly. High-fat diet alone, a model of pre-diabetes, while inducing impaired glucose tolerance and insulin resistance, does not cause the hyperglycemia characteristic of DM2 [52]. To induce DM2, mice were fasted overnight on week 4 of the high-fat diet and injected i.p. with nicotinamide (NA; 240 mg/kg, #72340, Sigma, St. Louis, MO, USA) and streptozotocin (STZ; 100 mg/kg, i.p., #1621, Tocris, Minneapolis, MN, USA) 15 min later; this was repeated 48 h later, as previously described [24]. STZ treatment alone (which mimics type 1 diabetes) results in loss of pancreatic beta cells; however, addition of NA to the STZ regimen prevents loss of beta cells and is an established model of DM2 [53]. After 6 weeks on a high-fat diet, and 2 weeks following NA/STZ injections, mice were fasted overnight and subjected to glucose tolerance testing (GTT) to verify induction of type 2 diabetes. Blood glucose was measured just prior to the ingestion of glucose (2 g/kg, oral gavage), and every 15–30 min for 2 h. Following GTT, mice were allowed to recover for a week before surgery. This is the same stroke model used in our previous study demonstrating that pre-stroke administration of tAUCB is protective against brain injury in diabetic stroke [24]. Additionally, this model is suited to the hypothesis we tested. Specifically, diabetes can exacerbate ischemic injury by multiple mechanisms that develop over time, including acute, subacute and chronic mechanisms. For example, hyperglycemia can exacerbate ischemic injury even in the absence of chronic diabetes; i.e., acute hyperglycemia due to stress, drugs, etc. This, however, is a different mechanism than stroke injury exacerbation due to chronic inflammation or chronic diabetic microangiopathy. Our study was designed to investigate the latter mechanism, where microvascular and inflammatory changes develop slowly over time. The 7-week time window provides sufficient time for the development of chronic microvascular and inflammatory changes, without being prohibitively long; for example, compared to high-fat diet alone, which requires at least 6 months of feeding [54].

### 4.3. Non-Diabetic Mice

Non-diabetic mice were maintained on a diet of regular chow (13% fat) (PicoLab Laboratory Rodent Diet, 5LOD, LabDiet, St. Louis, MO, USA).

### 4.4. Middle Cerebral Artery Occlusion (MCAO)

Transient 60 min focal cerebral ischemia was induced using the intraluminal MCAO technique previously described [24]. Briefly, MCAO was induced in isoflurane-anesthetized mice by insertion of a silicone-coated 6-0 nylon monofilament into the right internal carotid artery until MCA flow was reduced to less than 30% of baseline for 60 min, following which the occluding filament was withdrawn, allowing for reperfusion. Vehicle (0.5% PEG 400 in saline) or tAUCB (4-[[*trans*-4-[[(tricyclo[3.3.1.13,7]dec-1-ylamino)carbonyl]amino]cyclohexyl]oxy]-benzoic acid; 2 mg/kg; #16568, Cayman Chemical, Ann Arbor, MI, USA) were administered i.p. 30 min into vessel occlusion. Mice were monitored with laser Doppler and rectal temperature probes. Body temperature was maintained at 36 ± 0.5 °C. Mice were allowed to recover and observed for 24 h, at which time they were anesthetized with isoflurane, blood collected, tail glucose measured, and brain removed for infarct size assessment following sacrifice by decapitation. Mice (non-diabetic and diabetic) were 16–17 weeks of age at time of MCAO.

### 4.5. Brain Infarct Size

Infarct size in cerebral cortex, caudate putamen and total hemisphere was measured at 24 h after MCAO in 2-mm thick coronal brain sections using 2,3,5-triphenyltetrazolium chloride (TTC) staining and digital image analysis (SigmaScan Pro 5.0, Aspire Software, Ashburn, VA, USA) as previously described [24]. To account for edema, infarcted area was estimated by subtracting the uninfarcted region in ipsilateral hemisphere from the contralateral hemisphere, and expressing infarct volume as a percentage of the contralateral hemisphere.

### 4.6. Plasma Eicosanoid Quantification

#### 4.6.1. Chemicals and Reagents

Glass tubes and DMSO were from Fisher Scientific (Fairlawn, NJ, USA). Glacial acetic acid and phosphoric acid were obtained from J.T. Baker (Phillipsburg, NJ, USA). Ethyl acetate and sample filters were from EMD Millipore, a subdivision of Merck KGaA, Darmstadt, Germany. Hexane UV, methanol and water were purchased from Burdick and Jackson (Muskegon, MI, USA). Butylated hydroxytolulene (BHT), triphenyl phosphine (TPP) and indomethacin were from Sigma Aldrich (St. Louis, MO, USA) The sample vials were obtained from Sun Sri (a subdivision of Fisher Scientific, Rockwood, TN, USA). Oasis HLB cartridges were from Waters (Milford, MA, USA). Lipid standards and internal standards were purchased from Cayman Chemical company (Ann Arbor, MI, USA). Biotage PRESSURE+ 48 Positive Pressure manifold (Biotage, Uppsala, Sweden). EDTA mouse plasma were from Innovative Research (Novi, MI, USA).

#### 4.6.2. Preparation of Eicosanoid Samples and Calibrators

Samples were prepared as follows: a 60 mg Oasis HLB cartridge was pre-equilibrated with 1 mL of methanol with 0.2 mg/mL TPP, followed by 1 mL of 0.025% phosphoric acid-10% methanol. Plasma was centrifuged briefly, and 0.1 mL was diluted with 0.9 mL of 0.025% phosphoric acid. Then, 10 µL of anti-oxidant solution consisting of 0.2 mg/mL BHT, 2 mg/mL TPP and 2 mg/mL indomethacin in methanol:ethanol 1:1 was added. Subsequently, 1 ng of internal standard mix consisting of 1 ng of each of the following—d_8_-15 HETE, d_6_-20 HETE, d_8_-14,15 EET (or d_11_-14,15 EET depending on commercial availability), and d_11_-14,15 DHET—was added. Diluted samples were then loaded onto the pre-equilibrated cartridges by gravity with 3–5 psi positive pressure only in the case of a clog. Cartridges were washed with 2 mL of 0.025% phosphoric acid-10% methanol solution. Cartridges were then dried with 3–5 psi nitrogen gas on the positive pressure unit for 15 min. After drying, the cartridge was eluted sequentially with 3 mL of ethyl acetate, followed by 2 mL of hexane:ethyl acetate and finally with 1 mL of hexane. Then, 20 µL of trap solution consisting of 2% DMSO in methanol was added to the top of the combined eluates. The eluates were dried under vacuum for 35 min at 35 °C. The tube walls were washed with 1 mL of hexane and the sample was immediately re-dried for approximately 7 min till just dry and then solubilized in 60 µL of 45:55 (*v*/*v*) acetonitrile:water, placed in sample vials with inserts and analyzed my LC-MS/MS. The injection volume was 30 µL. An un-extracted standard curve was run, area ratios were plotted and unknowns were determined using the slopes. A spiked and naïve control plasma prepared from commercial plasma were run routinely to confirm response and calibration. Plasma samples were obtained from mice by investigators using EDTA and were pre-treated with antioxidant mix 10 µL per ml before being frozen at −80 until analysis, freeze thaw was avoided.

#### 4.6.3. LC-MS/MS Analysis for Eicosanoid Metabolites

DHETs, HETEs and EETs levels were analyzed using a 5500 Q-TRAP hybrid/triple quadrupole linear ion trap mass spectrometer (Applied Biosystems, Foster City, CA, USA) with electrospray ionization (ESI) in negative mode. The mass spectrometer was interfaced to a Shimadzu (Columbia, MD, USA) SIL-20AC XR auto-sampler followed by 2 LC-20AD XR LC pumps and analysis on an Applied Biosystems/SCIEX Q5500 instrument (Foster City, CA, USA). The instrument was operated with the following settings: source voltage −4000 kV, GS1 40, GS2 40, CUR 35, TEM 450 and CAD gas HIGH. The scheduled MRM transitions monitored with a 2 min window are presented in Table 1. Compounds were infused individually, and instrument parameters optimized for each MRM transition. The gradient mobile phase was delivered at a flow rate of 0.5 mL per min, and consisted of two solvents—A: 0.05% acetic acid in water; and B: 0.05% acetic acid in acetonitrile. Initial concentration of solvent B was 45%, this was held for 0.1 min before being increased to 60% over 5 min, then increased to 62.5% over 9 min, followed by an increase to 95% over 2 min, held at 95% for 2 min, decreased to start conditions of 45% B over 0.5 min and then equilibrated for 5.5 min. The Betabasic-18 100 × 2, 3 µ column was kept at 40 °C using a Shimadzu CTO-20AC column oven. Data were acquired using Analyst 1.5.1 software and analyzed using MultiQuant 3.0.1 software (both SCIEX, Framingham, MA, USA). The standard curves were from 0 to 1000 pg/sample and the limit of quantification was 10 pg per sample, except for 19 HETE and 20 HETE, where the limit of quantification was 25 pg per sample, where the relative standard deviation was less than 20%.

### 4.7. Statistical Analysis

Data are expressed as mean ± SEM. Groups were compared by *t*-test for two groups or two-way ANOVA with post hoc Sidak’s multiple comparisons test or repeated measured one-way ANOVA with post hoc Tukey’s multiple comparisons test as indicated using GraphPad Prism 9.0.2 (GraphPad Software, Inc., La Jolla, CA, USA). Differences are reported as significant at *p* < 0.05.

## Figures and Tables

**Table 1:** MRM parameter for the analysis of arachidonic acid metabolites.

Compound	RetentionTime (min)	Q1 Mass	Q3 Mass	DP	EP	CE	CXP
14,15-EET	7.45	319	219	−120	−10	−18	−11
11,12-EET	8.14	319	208	−80	−10	−18	−9
8,9-EET	8.50	319	68.9	−90	−10	−26	−11
5,6-EET	8.83	319	191	−85	−10	−16	−7
d_8_ 14,15-EET	7.30	327	226	−95	−10	−18	−9
5-HETE	6.90	319.2	115	−100	−10	−20	−9
11-HETE	6.08	319.2	167	−75	−10	−24	−3
12-HETE	6.31	319.2	179	−105	−10	−20	−3
15-HETE	5.74	319.2	219	−115	−10	−20	−9
18-HETE	4.96	319.2	261	−65	−10	−22	−9
19-HETE	4.62	319.2	231	−100	−10	−22	−7
20-HETE	4.80	319.1	289	−130	−10	−26	−11
d_8_ 15-HETE	5.6	327	226	−85	−10	−20	−9
d_6_ 20-HETE	4.7	325	281	−75	−10	−24	−9
14,15-DHET	3.6	337	207	−100	−10	−26	−3
11,12-DHET	4.00	337	167	−90	−10	−28	−11
8,9-DHET	4.40	337	127	−75	−10	−30	−9
5,6-DHET	4.90	337	145	−115	−10	−24	−9
d_11_ 14,15-DHET	3.60	348	207	−120	−10	−40	−15

## Data Availability

Not applicable.
